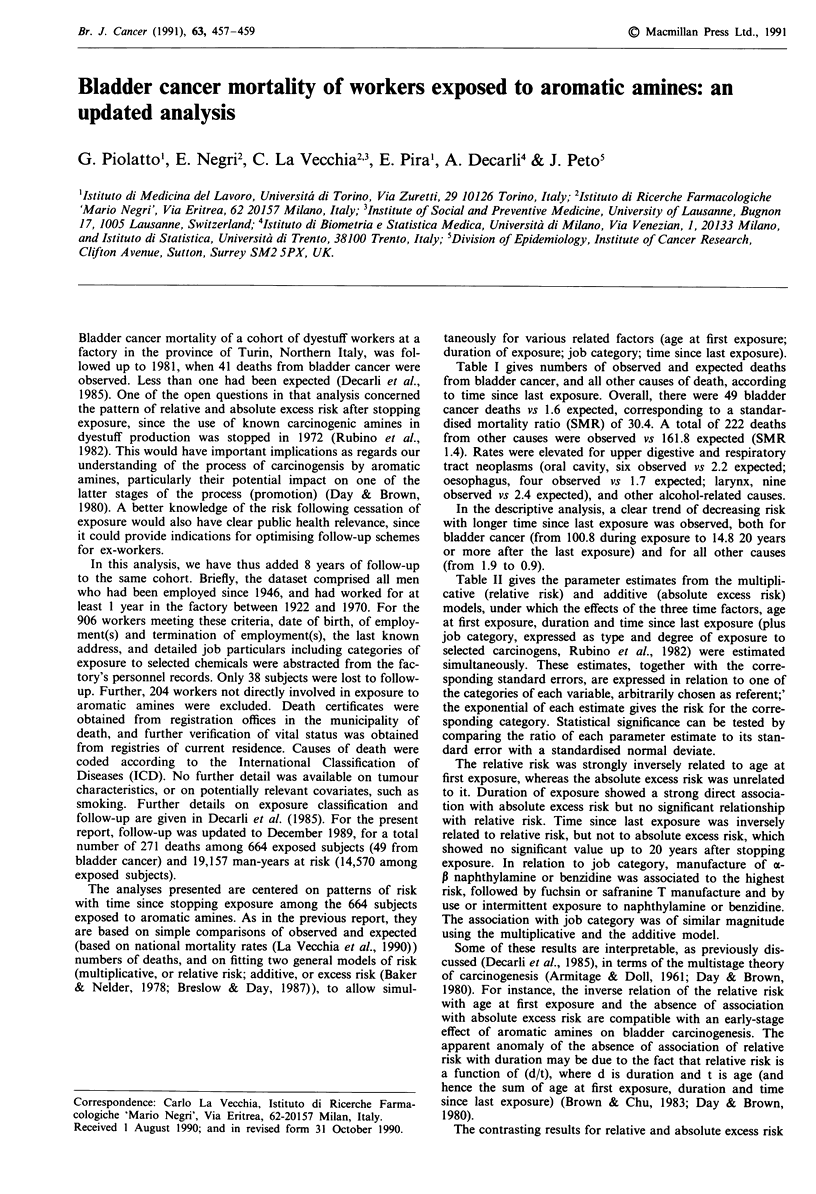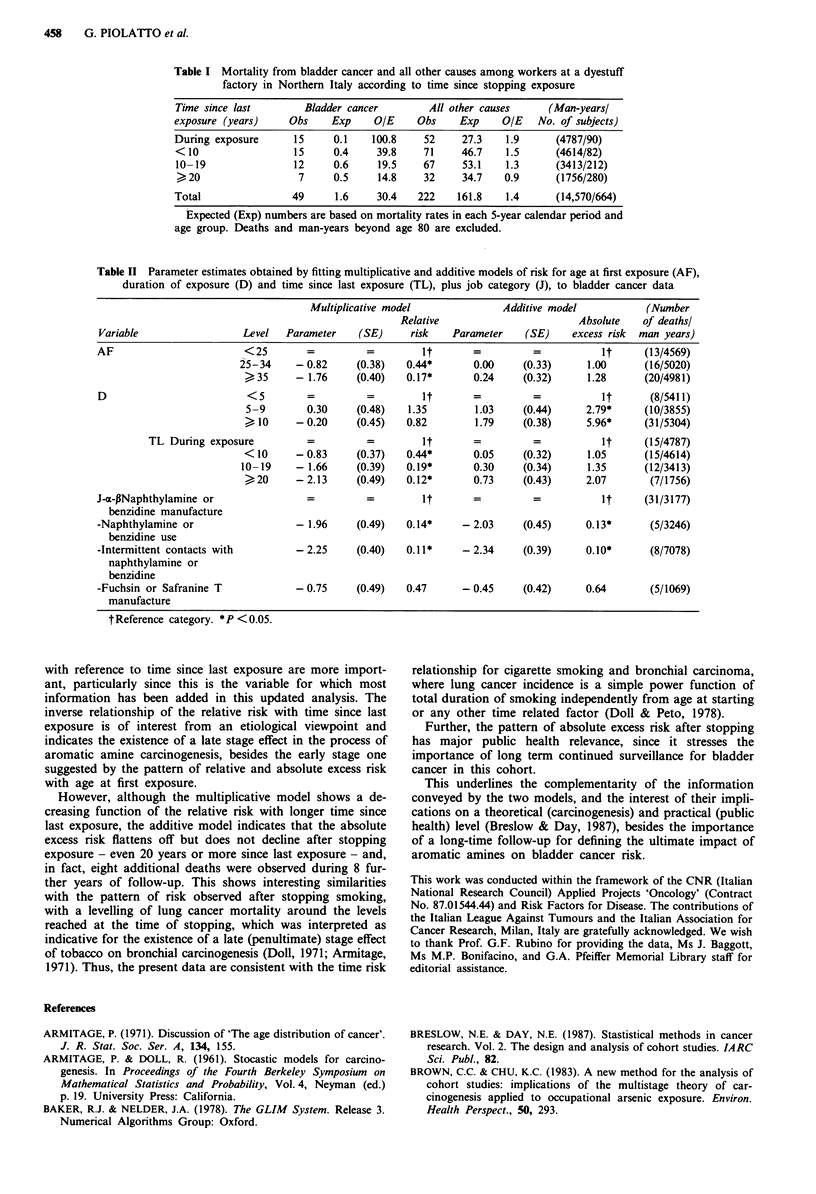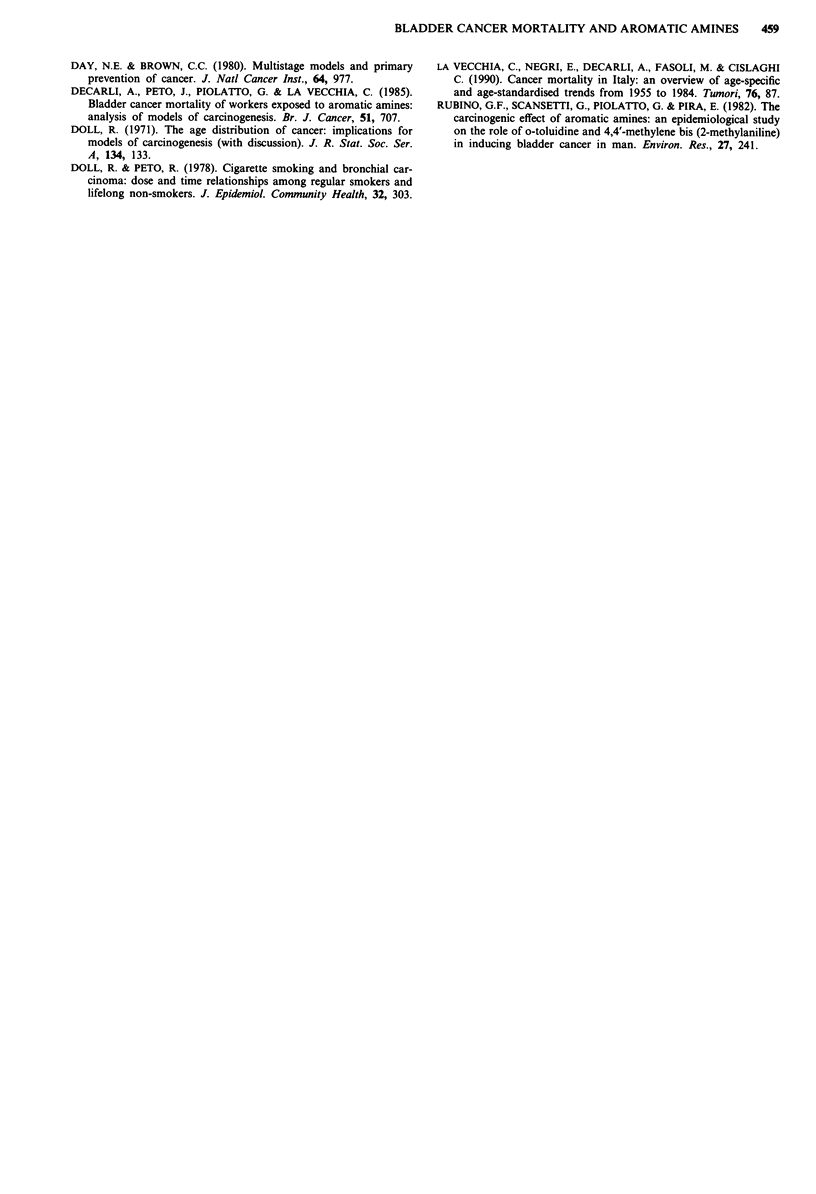# Bladder cancer mortality of workers exposed to aromatic amines: an updated analysis.

**DOI:** 10.1038/bjc.1991.106

**Published:** 1991-03

**Authors:** G. Piolatto, E. Negri, C. La Vecchia, E. Pira, A. Decarli, J. Peto

**Affiliations:** Istituto di Medicina del Lavoro, Universitá di Torino, Italy.


Br. J. Cancer (1991), 63, 457–459 © Macmillan Press Ltd., 1991

### Bladder cancer mortality of workers exposed to aromatic amines: an updated analysis

G. Piolatto[1], E. Negri[2], C. La Vecchia[2,3], E. Pira[1], A. Decarli[4] & J. Peto[5]

[1]*Istituto di Medicina del Lavoro, Universitá di Torino, Via Zuretti, 29 10126 Torino, Italy;* [2]*Istituto di Ricerche Farmacologiche 'Mario Negri', Via Eritrea, 62 20157 Milano, Italy;* [3]*Institute of Social and Preventive Medicine, University of Lausanne, Bugnon 17, 1005 Lausanne, Switzerland;* [4]*Istituto di Biometria e Statistica Medica, Università di Milano, Via Venezian, 1, 20133 Milano, and Istituto di Statistica, Università di Trento, 38100 Trento, Italy;* [5]*Division of Epidemiology, Institute of Cancer Research, Clifton Avenue, Sutton, Surrey SM2 5PX, UK.*

Bladder cancer mortality of a cohort of dyestuff workers at a factory in the province of Turin, Northern Italy, was followed up to 1981, when 41 deaths from bladder cancer were observed. Less than one had been expected (Decarli *et al.*, 1985). One of the open questions in that analysis concerned the pattern of relative and absolute excess risk after stopping exposure, since the use of known carcinogenic amines in dyestuff production was stopped in 1972 (Rubino *et al.*, 1982). This would have important implications as regards our understanding of the process of carcinogensis by aromatic amines, particularly their potential impact on one of the latter stages of the process (promotion) (Day & Brown, 1980). A better knowledge of the risk following cessation of exposure would also have clear public health relevance, since it could provide indications for optimising follow-up schemes for ex-workers.

In this analysis, we have thus added 8 years of follow-up to the same cohort. Briefly, the dataset comprised all men who had been employed since 1946, and had worked for at least 1 year in the factory between 1922 and 1970. For the 906 workers meeting these criteria, date of birth, of employment(s) and termination of employment(s), the last known address, and detailed job particulars including categories of exposure to selected chemicals were abstracted from the factory's personnel records. Only 38 subjects were lost to follow-up. Further, 204 workers not directly involved in exposure to aromatic amines were excluded. Death certificates were obtained from registration offices in the municipality of death, and further verification of vital status was obtained from registries of current residence. Causes of death were coded according to the International Classification of Diseases (ICD). No further detail was available on tumour characteristics, or on potentially relevant covariates, such as smoking. Further details on exposure classification and follow-up are given in Decarli *et al.* (1985). For the present report, follow-up was updated to December 1989, for a total number of 271 deaths among 664 exposed subjects (49 from bladder cancer) and 19,157 man-years at risk (14,570 among exposed subjects).

The analyses presented are centered on patterns of risk with time since stopping exposure among the 664 subjects exposed to aromatic amines. As in the previous report, they are based on simple comparisons of observed and expected (based on national mortality rates (La Vecchia *et al.*, 1990)) numbers of deaths, and on fitting two general models of risk (multiplicative, or relative risk; additive, or excess risk (Baker & Nelder, 1978; Breslow & Day, 1987)), to allow simultaneously for various related factors (age at first exposure; duration of exposure; job category; time since last exposure).

Table I gives numbers of observed and expected deaths from bladder cancer, and all other causes of death, according to time since last exposure. Overall, there were 49 bladder cancer deaths *vs* 1.6 expected, corresponding to a standardised mortality ratio (SMR) of 30.4. A total of 222 deaths from other causes were observed *vs* 161.8 expected (SMR 1.4). Rates were elevated for upper digestive and respiratory tract neoplasms (oral cavity, six observed *vs* 2.2 expected; oesophagus, four observed *vs* 1.7 expected; larynx, nine observed *vs* 2.4 expected), and other alcohol-related causes.

In the descriptive analysis, a clear trend of decreasing risk with longer time since last exposure was observed, both for bladder cancer (from 100.8 during exposure to 14.8 20 years or more after the last exposure) and for all other causes (from 1.9 to 0.9).

Table II gives the parameter estimates from the multiplicative (relative risk) and additive (absolute excess risk) models, under which the effects of the three time factors, age at first exposure, duration and time since last exposure (plus job category, expressed as type and degree of exposure to selected carcinogens, Rubino *et al.*, 1982) were estimated simultaneously. These estimates, together with the corresponding standard errors, are expressed in relation to one of the categories of each variable, arbitrarily chosen as referent;' the exponential of each estimate gives the risk for the corresponding category. Statistical significance can be tested by comparing the ratio of each parameter estimate to its standard error with a standardised normal deviate.

The relative risk was strongly inversely related to age at first exposure, whereas the absolute excess risk was unrelated to it. Duration of exposure showed a strong direct association with absolute excess risk but no significant relationship with relative risk. Time since last exposure was inversely related to relative risk, but not to absolute excess risk, which showed no significant value up to 20 years after stopping exposure. In relation to job category, manufacture of α-β naphthylamine or benzidine was associated to the highest risk, followed by fuchsin or safranine T manufacture and by use or intermittent exposure to naphthylamine or benzidine. The association with job category was of similar magnitude using the multiplicative and the additive model.

Some of these results are interpretable, as previously discussed (Decarli *et al.*, 1985), in terms of the multistage theory of carcinogenesis (Armitage & Doll, 1961; Day & Brown, 1980). For instance, the inverse relation of the relative risk with age at first exposure and the absence of association with absolute excess risk are compatible with an early-stage effect of aromatic amines on bladder carcinogenesis. The apparent anomaly of the absence of association of relative risk with duration may be due to the fact that relative risk is a function of (d/t), where d is duration and t is age (and hence the sum of age at first exposure, duration and time since last exposure) (Brown & Chu, 1983; Day & Brown, 1980).

The contrasting results for relative and absolute excess risk

Correspondence: Carlo La Vecchia, Istituto di Ricerche Farmacologiche 'Mario Negri', Via Eritrea, 62-20157 Milan, Italy.
Received 1 August 1990; and in revised form 31 October 1990.

**Table I** Mortality from bladder cancer and all other causes among workers at a dyestuff factory in Northern Italy according to time since stopping exposure

| *Time since last exposure (years)* | *Bladder cancer Obs* | *Exp* | *O/E* | *All other causes Obs* | *Exp* | *O/E* | *(Man-years/ No. of subjects)* |
|---|---|---|---|---|---|---|---|
| During exposure | 15 | 0.1 | 100.8 | 52 | 27.3 | 1.9 | (4787/90) |
| <10 | 15 | 0.4 | 39.8 | 71 | 46.7 | 1.5 | (4614/82) |
| 10–19 | 12 | 0.6 | 19.5 | 67 | 53.1 | 1.3 | (3413/212) |
| ⩾20 | 7 | 0.5 | 14.8 | 32 | 34.7 | 0.9 | (1756/280) |
| Total | 49 | 1.6 | 30.4 | 222 | 161.8 | 1.4 | (14,570/664) |

Expected (Exp) numbers are based on mortality rates in each 5-year calendar period and age group. Deaths and man-years beyond age 80 are excluded.

**Table II** Parameter estimates obtained by fitting multiplicative and additive models of risk for age at first exposure (AF), duration of exposure (D) and time since last exposure (TL), plus job category (J), to bladder cancer data

| *Variable* | *Level* | *Multiplicative model Parameter* | *(SE)* | *Relative risk* | *Additive model Parameter* | *(SE)* | *Absolute excess risk* | *(Number of deaths/ man years)* |
|---|---|---|---|---|---|---|---|---|
| AF | <25 | = | = | 1† | = | = | 1† | (13/4569) |
| | 25–34 | − 0.82 | (0.38) | 0.44* | 0.00 | (0.33) | 1.00 | (16/5020) |
| | ⩾35 | − 1.76 | (0.40) | 0.17* | 0.24 | (0.32) | 1.28 | (20/4981) |
| D | <5 | = | = | 1† | = | = | 1† | (8/5411) |
| | 5–9 | 0.30 | (0.48) | 1.35 | 1.03 | (0.44) | 2.79* | (10/3855) |
| | ⩾10 | − 0.20 | (0.45) | 0.82 | 1.79 | (0.38) | 5.96* | (31/5304) |
| TL | During exposure | = | = | 1† | = | = | 1† | (15/4787) |
| | <10 | − 0.83 | (0.37) | 0.44* | 0.05 | (0.32) | 1.05 | (15/4614) |
| | 10–19 | − 1.66 | (0.39) | 0.19* | 0.30 | (0.34) | 1.35 | (12/3413) |
| | ⩾20 | − 2.13 | (0.49) | 0.12* | 0.73 | (0.43) | 2.07 | (7/1756) |
| J-α-βNaphthylamine or benzidine manufacture | | = | = | 1† | = | = | 1† | (31/3177) |
| -Naphthylamine or benzidine use | | − 1.96 | (0.49) | 0.14* | − 2.03 | (0.45) | 0.13* | (5/3246) |
| -Intermittent contacts with naphthylamine or benzidine | | − 2.25 | (0.40) | 0.11* | − 2.34 | (0.39) | 0.10* | (8/7078) |
| -Fuchsin or Safranine T manufacture | | − 0.75 | (0.49) | 0.47 | − 0.45 | (0.42) | 0.64 | (5/1069) |

†Reference category. *$P < 0.05$.

with reference to time since last exposure are more important, particularly since this is the variable for which most information has been added in this updated analysis. The inverse relationship of the relative risk with time since last exposure is of interest from an etiological viewpoint and indicates the existence of a late stage effect in the process of aromatic amine carcinogenesis, besides the early stage one suggested by the pattern of relative and absolute excess risk with age at first exposure.

However, although the multiplicative model shows a decreasing function of the relative risk with longer time since last exposure, the additive model indicates that the absolute excess risk flattens off but does not decline after stopping exposure – even 20 years or more since last exposure – and, in fact, eight additional deaths were observed during 8 further years of follow-up. This shows interesting similarities with the pattern of risk observed after stopping smoking, with a levelling of lung cancer mortality around the levels reached at the time of stopping, which was interpreted as indicative for the existence of a late (penultimate) stage effect of tobacco on bronchial carcinogenesis (Doll, 1971; Armitage, 1971). Thus, the present data are consistent with the time risk relationship for cigarette smoking and bronchial carcinoma, where lung cancer incidence is a simple power function of total duration of smoking independently from age at starting or any other time related factor (Doll & Peto, 1978).

Further, the pattern of absolute excess risk after stopping has major public health relevance, since it stresses the importance of long term continued surveillance for bladder cancer in this cohort.

This underlines the complementarity of the information conveyed by the two models, and the interest of their implications on a theoretical (carcinogenesis) and practical (public health) level (Breslow & Day, 1987), besides the importance of a long-time follow-up for defining the ultimate impact of aromatic amines on bladder cancer risk.

This work was conducted within the framework of the CNR (Italian National Research Council) Applied Projects 'Oncology' (Contract No. 87.01544.44) and Risk Factors for Disease. The contributions of the Italian League Against Tumours and the Italian Association for Cancer Research, Milan, Italy are gratefully acknowledged. We wish to thank Prof. G.F. Rubino for providing the data, Ms J. Baggott, Ms M.P. Bonifacino, and G.A. Pfeiffer Memorial Library staff for editorial assistance.

### References

ARMITAGE, P. (1971). Discussion of 'The age distribution of cancer'. *J. R. Stat. Soc. Ser. A*, **134**, 155.

ARMITAGE, P. & DOLL, R. (1961). Stocastic models for carcinogenesis. In *Proceedings of the Fourth Berkeley Symposium on Mathematical Statistics and Probability*, Vol. 4, Neyman (ed.) p. 19. University Press: California.

BAKER, R.J. & NELDER, J.A. (1978). *The GLIM System*. Release 3. Numerical Algorithms Group: Oxford.

BRESLOW, N.E. & DAY, N.E. (1987). Stastistical methods in cancer research. Vol. 2. The design and analysis of cohort studies. *IARC Sci. Publ.*, **82**.

BROWN, C.C. & CHU, K.C. (1983). A new method for the analysis of cohort studies: implications of the multistage theory of carcinogenesis applied to occupational arsenic exposure. *Environ. Health Perspect.*, **50**, 293.

DAY, N.E. & BROWN, C.C. (1980). Multistage models and primary prevention of cancer. *J. Natl Cancer Inst.*, **64**, 977.

DECARLI, A., PETO, J., PIOLATTO, G. & LA VECCHIA, C. (1985). Bladder cancer mortality of workers exposed to aromatic amines: analysis of models of carcinogenesis. *Br. J. Cancer*, **51**, 707.

DOLL, R. (1971). The age distribution of cancer: implications for models of carcinogenesis (with discussion). *J. R. Stat. Soc. Ser. A*, **134**, 133.

DOLL, R. & PETO, R. (1978). Cigarette smoking and bronchial carcinoma: dose and time relationships among regular smokers and lifelong non-smokers. *J. Epidemiol. Community Health*, **32**, 303.

LA VECCHIA, C., NEGRI, E., DECARLI, A., FASOLI, M. & CISLAGHI C. (1990). Cancer mortality in Italy: an overview of age-specific and age-standardised trends from 1955 to 1984. *Tumori*, **76**, 87.

RUBINO, G.F., SCANSETTI, G., PIOLATTO, G. & PIRA, E. (1982). The carcinogenic effect of aromatic amines: an epidemiological study on the role of o-toluidine and 4,4′-methylene bis (2-methylaniline) in inducing bladder cancer in man. *Environ. Res.*, **27**, 241.